# Automatic graphene transfer system for improved material quality and efficiency

**DOI:** 10.1038/srep21676

**Published:** 2016-02-10

**Authors:** Alberto Boscá, Jorge Pedrós, Javier Martínez, Tomás Palacios, Fernando Calle

**Affiliations:** 1 Instituto de Sistemas Optoelectrónicos y Microtecnología, Universidad Politécnica de Madrid, Madrid, 28040, Spain; 2 Dpto. de Ingeniería Electrónica, E.T.S.I de Telecomunicación, Universidad Politécnica de Madrid, Madrid, 28040, Spain; 3 Campus de Excelencia Internacional, Campus Moncloa UCM-UPM, Madrid, 28040, Spain; 4 Dpto. de Ciencia de Materiales, E.T.S.I de Caminos, Canales y Puertos, Universidad Politécnica de Madrid, Madrid, 28040, Spain; 5 Department of Electrical Engineering and Computer Science, Massachusetts Institute of Technology, Cambridge, Massachusetts, 02139, USA

## Abstract

In most applications based on chemical vapor deposition (CVD) graphene, the transfer from the growth to the target substrate is a critical step for the final device performance. Manual procedures are time consuming and depend on handling skills, whereas existing automatic roll-to-roll methods work well for flexible substrates but tend to induce mechanical damage in rigid ones. A new system that automatically transfers CVD graphene to an arbitrary target substrate has been developed. The process is based on the all-fluidic manipulation of the graphene to avoid mechanical damage, strain and contamination, and on the combination of capillary action and electrostatic repulsion between the graphene and its container to ensure a centered sample on top of the target substrate. The improved carrier mobility and yield of the automatically transferred graphene, as compared to that manually transferred, is demonstrated by the optical and electrical characterization of field-effect transistors fabricated on both materials. In particular, 70% higher mobility values, with a 30% decrease in the unintentional doping and a 10% strain reduction are achieved. The system has been developed for lab-scale transfer and proved to be scalable for industrial applications.

Graphene has been shown to be an excellent material for optical^1^, electronic^2^, plasmonic^3^ and energy^4^ applications. Among the different techniques to obtain graphene, chemical vapor deposition (CVD) on a metal substrate presents the best tradeoff between material quality and industrial scalability. However, for most applications a transparent and/or insulating substrate is needed. Therefore, the graphene layer requires to be transferred from the metal catalyst to the target substrate. For this purpose, several procedures have been developed.

A manual method for graphene transfer is widely used^5^,^6^. It mainly consists on protecting the graphene layer with a polymer, etching chemically the metal catalyst in an etchant solution to release the film in the liquid, rinsing with deionized water (DIW), and finally transferring it to the target substrate. A lot of optimization work has been done to improve this method in order to minimize the amount of residues in graphene^7^,^8^,^9^,^10^, select the transfer region on the target substrate^11^,^12^,^13^,^14^, recover the growth substrate for its reutilization^15^,^16^,^17^, or to enhance the film handling to minimize the mechanical damage on the graphene, either by adding a rigid carrier^18^,^19^,^20^,^21^,^22^,^23^ or by using capillarity effects^24^ or electrostatic forces^25^,^26^ that restrict the type of target substrate. Most of these methods are simple and cost-effective, but they are time-consuming and require handling skills, affecting the yield and reproducibility of the transfer process.

Despite of these drawbacks, the only existing automatic methods are based on roll-to-roll transfer processes^27^,^28^. They provide a scalable method to obtain graphene film attached to flexible substrates, but when applied to rigid substrates such as SiO_2_/Si wafers they tend to cause undesired mechanical defects on graphene films, which considerably degrade their electrical properties^20^. Therefore, an automatic transfer system for arbitrary substrates is still needed.

In this work we demonstrate a new system that automatically transfers the graphene layer to an arbitrary target substrate, avoiding the need of an operator and the waiting times of a typical manual transfer process. Furthermore, we demonstrate the improved carrier mobility and yield of the automatically transferred graphene, as compared to that manually transferred, as shown by the optical and electrical characterization of graphene field-effect transistors (GFETs) fabricated on both kind of materials. The system reported has been developed for lab-scale transfer, but it is fully scalable and, thus, suitable for industrial applications.

## Results and Discussion

### Transfer system design

**Figure 1:**
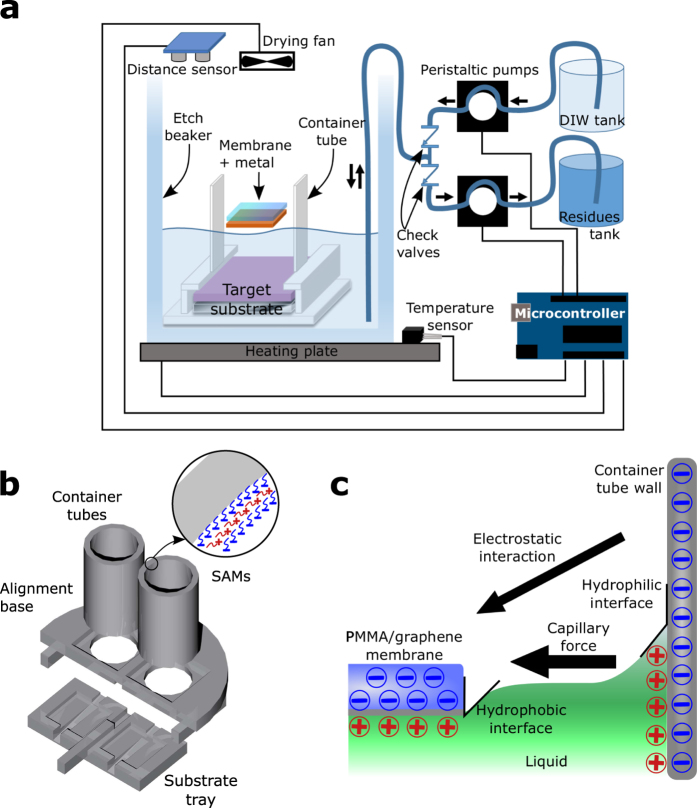
(**a**) Schematic of the automatic transfer system components and their connections. A microcontroller communicates with all the sensors and actuators. The liquid is forced to flow from the DIW tank to the etch beaker and from the latter to the residues tank by means of peristaltic pumps and check valves. The temperature is controlled by means of a heating plate and a temperature sensor. (**b**) PTFE holding structure for the automatic transfer system. The container tubes, mounted on the alignment base, keep the membrane centered during the whole process regarding the target substrate placed in the substrate tray underneath. The inset shows a detail of the stack of SAMs coating the inner wall of the container tube. (**c**) Schematic of the centering mechanism of the membrane inside the container tube between the graphene film and the SAM-treated inner wall of the tube due to capillary and electrostatic repulsion forces.

The automatic transfer system is described schematically in Fig. 1. The polymer-protected graphene layer on a metal foil and the substrate are introduced into a polytetrafluoroethylene (PTFE) holding structure contained in a regular borosilicate glass beaker. A programmable control system allows to select the temperature, the liquid flow, and the drying conditions during the process.

The PTFE holding structure has been specifically designed to assist the automatic process (Fig. 1b). The chosen material avoids degradation and contamination issues. The structure consists of three parts: the substrate tray, where the target substrates are loaded, the container tubes, that ensure the centered position of the graphene film, and the alignment base, that fixes the position between the substrate tray and the container tubes. A two-substrate loading system was designed to demonstrate the parallel processing capability of the system and to optimize the space inside the beaker. However, the system can be scaled-up, both in the size of the substrates and in the number of simultaneous substrates. A scalability test has been successfully performed for 4” target substrates (see Fig. S1 in the Supplementary Information) that paves the way for industrial applications. A video of the scaled-up process is available online. A special treatment of the inner surface of the container tubes has been carried out in order to modify the PTFE properties to assist the automatic transfer process (inset in Fig. 1b). A stack of self-assembled monolayers (SAMs)^29^ is placed onto the inner walls of the container tubes (See Methods). This coating makes the PTFE surface hydrophilic (opposite to the hydrophobic character of the untreated material) and charges it negatively. The hydrophilicity prevents the liquid surface tension from damaging the graphene during the transfer process, as the hydrophobic behavior of graphene (contact angle CA = 93°)^30^ will generate a repulsive capillary force^31^ between the wall and the membrane. Since some metal catalysts, such as copper, can be slightly hydrophilic (CA = 86°)^30^, the electrostatic repulsion can be canceled out and the capillarity force reversed, resulting in uncentered samples during the initial transfer steps. Once the metal catalyst is etched away, the graphene layer inside the container tube is forced to stay centered due to the combined effect of the capillary action and the electrostatic repulsion force between the inner surface of the container tube and the protective polymer layer, both negatively charged (Fig. 1c). Due to ionic screening in the liquid, only the dry surface of the container tube interacts electrostatically with the floating membrane. It has been tested that inverting the order of the SAMs coating the container tube, and thus producing a positively charged surface, leads to unwanted graphene adhesion to the tube wall. Therefore, both, electrostatic repulsion and capillary action are needed for a successful transfer. As the target substrate is centered with respect to the container tube by the alignment base, this mechanism grants the final centered position of the transferred graphene onto the target substrate.

The control electronics consist of an Arduino UNO microcontroller that is used for connecting, timing and reading the other system modules. A serial connection to a PC is used for programming and logging the process.

Regarding the fluidics, two peristaltic pumps are used for introducing and removing liquid from the etch beaker. Chemical-resistant tubing connect the three containers of the liquid system: the etch beaker, and the DIW and the residues tanks. For the liquid flow, three states are available: idle, liquid introduction and liquid removal. By means of two check valves, the liquid can only flow from the DIW tank to the etch beaker and from the etch beaker to the residues tank, corresponding with the liquid introduction and liquid removal states in the peristaltic pumps system, respectively. The on/off pump conditions are controlled by means of relay switches.

A contactless, ultrasound-based distance sensor reads directly the liquid level inside the etch beaker, providing the feedback signal for controlling the fluidics and determining the filling state. This sensor does not disturb the liquid surface, and is reliable even when the liquid composition is changing, as opposed to infrared sensors. The distance sensor is mounted on a micropositioner that allows fine tuning the initial distance before running the automatic process. Minimum and maximum liquid levels are defined in order to keep the graphene constantly floating in the liquid inside the container tube without touching the target substrate underneath till the very last step of the transfer process. This is a key characteristic of the automatic transfer system, as compared to the manual transfer methods, where the sample is changed abruptly to different liquid solutions using an intermediate holding substrate. Thus, the all-fluidic manipulation prevents the graphene from any strain, damage, or contamination induced by the intermediate holding substrate.

The heating plate along with the temperature sensor form the temperature control unit. A proportional-integral-derivative controller is used for setting the temperature, being optimized for the working temperature range of the system (RT - 60 °C).

Finally, a fan positioned on top of the container tubes is activated during the last step of the transfer process to help drying the samples.

### Transfer process

Before starting the automatic system operation, the target substrate is loaded onto the substrate tray. For some cases, such as chemically-reactive target substrate, the substrate tray can be loaded onto the system just before the end of the process. This way, the target substrate will only contact pure DIW. For flexible substrates, such as thin PET films, a rigid holder should be used to fix the substrate to the substrate tray. For preparing the transfer process, the whole PTFE structure is introduced inside the etch beaker, with the alignment base and container tubes in place as shown in Fig. 1b. The beaker is manually filled with the etchant solution, and the graphene sample (with the graphene surface coated with a protective polymer layer) is carefully introduced inside the container tube, leaving it floating with the unprotected metal surface in contact with the etchant liquid. Marble’s reagent has been used as etchant and poly(methyl methacrylate) (PMMA) (950K 2.5% mass in chlorobenzene), spin-coated at 5000 r.p.m (0.25 *μ*m thickness), has been used as protective polymer. Finally, the distance sensor on top of the beaker is checked and readjusted if needed, and the tubing end is introduced at the bottom of the beaker. After this point, the system operation is fully automated with no need of operator intervention.

**Figure 2:**
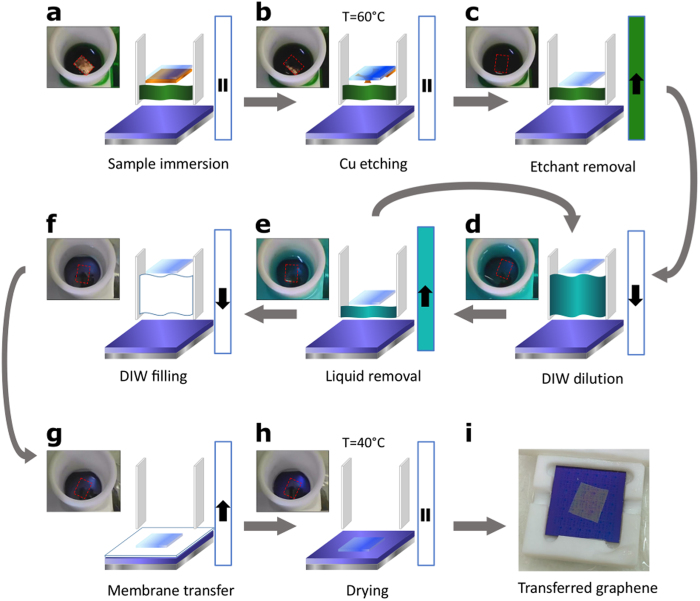
Process flow diagram of the automatic transfer. (**a**) The sample is introduced in the system containing the etching solution. (**b**) The temperature is set to 60 °C during 30 minutes for the Cu etching state. (**c**) Once the sample is completely free of Cu, the etchant solution is drained. Subsequent DIW dilutions (**d**) and liquid removal steps (**e**) are performed to remove any trace of the etchant solution. (**f**) After several iterations of steps (**c**,**d**), the sample is floating in pure DIW. (**g**) DIW is fully drained and the PMMA/graphene membrane is transferred to the target substrate. (**h**) The temperature is set to 40 °C during 20 minutes to remove the interstitial water between the graphene and the substrate. (**i**) Transferred 1 × 1 cm^2^ PMMA/graphene membrane to the 2 × 2 cm^2^ target substrate after the process. The photographs show the centered position of the membrane inside the container tube along the whole process (the dashed red line is a guide for the eye).

In Fig. 2 the series of steps in the automatic transfer process are detailed. After placing the graphene sample inside the tube (Fig. 2a) the temperature is raised to 60 °C and a 30 minute timer starts (Fig. 2b). This temperature ensures the complete substrate removal in the time interval set. As the metal is being etched, the self-centering mechanism of the membrane starts to act as detailed previously. Once the metal is fully etched, the PMMA/graphene film floats perfectly centered inside the container tube. Then, the peristaltic pumps are set to the liquid removal state until the minimum liquid level is reached (Fig. 2c). Afterwards, a set of diluting steps starts. First, DIW is introduced in the etch beaker using the liquid introduction pump state until the maximum liquid level is reached (Fig. 2d). Second, the diluted solution in the etch beaker is removed with the peristaltic pumps set to the liquid removal state until the minimum liquid level is reached (Fig. 2e). These two steps are repeated consecutively several times in order to remove the etchant ions from the final DIW. The liquid conductivity during these steps has been checked, requiring less than 2 hours to reach the conductivity of ultra-pure water (see Fig. S2 in the Supplementary Information). DIW consumption depends on the sample and target substrate sizes. For 1 × 1 cm^2^ membranes on 2 × 2 cm^2^ substrates, 400 ml of DIW are needed for the rinse, though a system design optimization could reduce this amount.

After the dilution loop, the critical part of the transfer process starts, filling the etch beaker with DIW up to the maximum liquid level (Fig. 2f), and then draining it completely (Fig. 2g). In this step, the PMMA/graphene film is softly transferred to the target substrate, in a centered position due to the action of the electrostatic repulsion forces. At this point, the sample lies on top of the target substrate, though a thin water layer separates the graphene from the substrate surface. In order to remove this water interlayer and have a good adhesion to the surface, the sample is heated up to 40 °C for 20 minutes while a perpendicular air flow generated with a fan helps to dry it out efficiently (Fig. 2h).

Once the automatic transfer process ends, the target substrate is unloaded from the system. In Fig. 2i, the final substrate with the transferred PMMA/graphene film is shown. With the conditions described above, the whole automatic transfer process lasts 2.5 hours, with no action needed from the operator during that time. The PMMA is then removed from the graphene using acetone or other solvent in the same way as in the manual transfer method. The cleanliness of the resulting graphene surface has been assessed by atomic force microscopy (see Fig. S3 in the Supplementary Information).

### Enhancement verification

In addition to the technological advantages inherent to an automated process (no handling skills required, good reproducibility, large throughput, etc.), an automatic transfer process is expected to provide better quality graphene in terms of cleanliness (i.e. lower amount of etchant residues) and structural integrity, since the manipulation of the graphene is done in a smoother way with no intermediate holding substrate involved. The usage of an intermediate substrate in the manual transfer may cause undesired effects, as a quick capture movement is needed to perform several steps. For example, the membrane is forced an unwanted tensile strain when raised from the liquid, as the membrane is partially floating while the liquid is perturbed. Other undesired effect may be wrinkle formation around etchant residues, that may promote the adhesion of trapped ions on the graphene surface. An explanatory schematic of both mechanisms can be found in Fig. S4 of the Supplementary Information.

**Figure 3:**
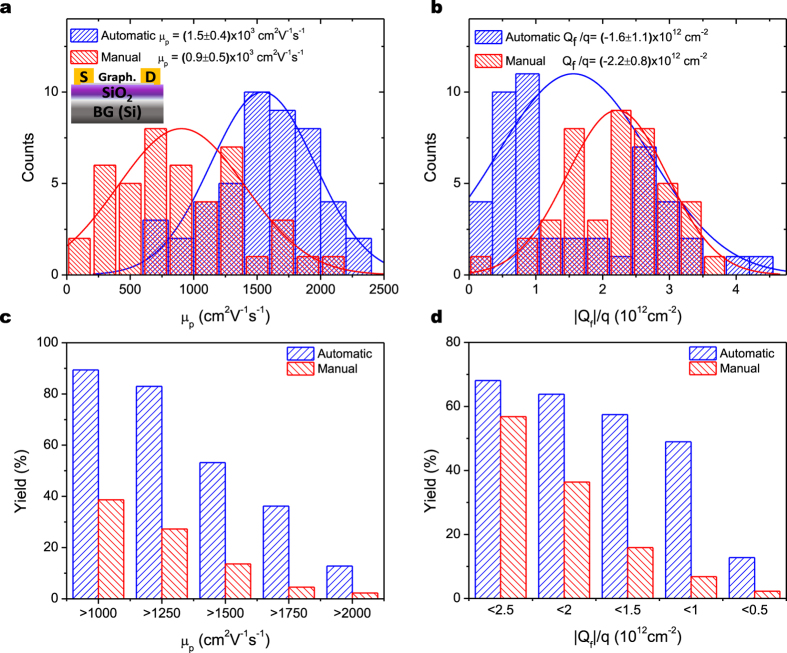
Histograms of the (**a**) mobility *μ* and (**b**) doping concentration 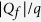 extracted from the GFET devices fabricated using automatically and manually transferred graphene. The inset in (**a**) shows the schematic of the GFET. The values of the magnitudes indicated correspond to the mean and standard deviation values of the histograms fitted to a normal distribution (lines). Yield of GFETs with (**c**) mobility and (**d**) doping values above a given threshold. Higher mean mobility and lower mean doping values, as well as much better yields, are observed for the devices fabricated on automatically transferred graphene.

In order to demonstrate the improvement of the properties in automatically-transferred graphene, the performance of GFETs fabricated on manually and automatically transferred samples was compared. Thus, two groups of samples from the same graphene material were processed with the same conditions (see Methods), except for the graphene transfer method: the first group was transferred manually following a method derived from Sun *et al.*^32^, whereas the second group was transferred using our automated transfer system. The graphene material used in both cases was commercially available CVD monolayer graphene on 25 *μ*m-thick Cu foil. The chosen target substrates are 2 × 2 cm^2^ pieces diced from a 300 nm-thick SiO_2_/Si 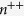 wafer, where Si is used as the back-gate (Fig. 3 inset).

For analyzing the quality of the transferred graphene, a physical model has been used for extracting relevant electric parameters from the GFET characteristics^33^. The two key parameters extracted are the mobility (*μ*) (Fig. 3a) and the unintended doping concentration or fixed charge density attached to the graphene (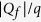) (Fig. 3b). The latter indicates the amount of residues adsorbed to graphene, which, in turn, increase the scattering and degrade the mobility. As the PMMA removal procedure is exactly the same for both automatically and manually transferred samples, differences in their doping level should be related to etchant rather than PMMA residues.

In the case of the mobility, a clear improvement is observed for the automatically transferred samples, with a 70% increase in the mobility mean value as compared to the manually transferred samples. This is consistent with the decrease of 30% in the unintended doping for the automatically transferred samples, indicating less adsorbed impurities than in the manual method and, thus, less scattering centers reducing the mobility of the carriers in the graphene film. These results confirm that the automatic method developed degrades less the electrical properties of the material. The values reported for the manual transfer are in good agreement with literature values for the same target substrate and graphene source^34^ (830 cm^2^V^−1^s^−1^). As discussed above, the gradual dilution procedure used in the automatic transfer process is likely to remove more efficiently the etchant from the graphene surface in contact with the liquid, thus reducing the unintentional doping concentration. Additionally, the all-fluidic manipulation of the graphene membrane in the automatic transfer process avoids the mechanical stress induced during the successive DIW rinse steps and the final transfer step to the target substrate of the manual method. Nonetheless, for both types of samples, the standard deviation in the mobility is similar, indicating that the dispersion may be related with the intrinsic properties of the commercial CVD-graphene used in all cases.

It is also important to point out that the automatic transfer method provides a much larger yield. The yields of the manual and automatic transfer processes, expressed as the fraction of devices with higher mobility or lower doping values than certain thresholds, are shown in Fig. 3c,d, respectively. The threshold values are ordered from less to more demanding, as the decreasing tendency indicates. In the case of the mobility, the increase in the yield for the automatically transferred devices compared with the manually transferred ones goes from more than two-fold for the less demanding threshold value (1000 cm^2^V^−1^s^−1^) to eight-fold for the most demanding threshold value (2000 cm^2^V^−1^s^−1^). A similar behavior is found for the unintentional doping level, with a seven-fold increase in the yield for the automatically transferred devices compared with the manually transferred ones for doping concentrations as low as 10^12^ cm^−2^.

Raman spectroscopy mapping has been performed in the channel area of several GFETs fabricated on both automatically and manually transferred graphene samples in order to evaluate the amount of strain and doping present in each kind of samples and correlate them with the results obtained from the electrical characterization of the devices. The analysis is based on the correlation of the frequencies of the Raman modes G (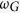) and 2D (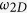) and their decomposition on a basis of strain- and charge-sensitivity^35^,^36^,^37^. The state for unstrained and charge-neutral graphene from Lee *et al.*^35^ was corrected for the 532 nm wavelength excitation used in the Raman measurements using frequency-shift values extracted from the literature^38^.

**Figure 4:**
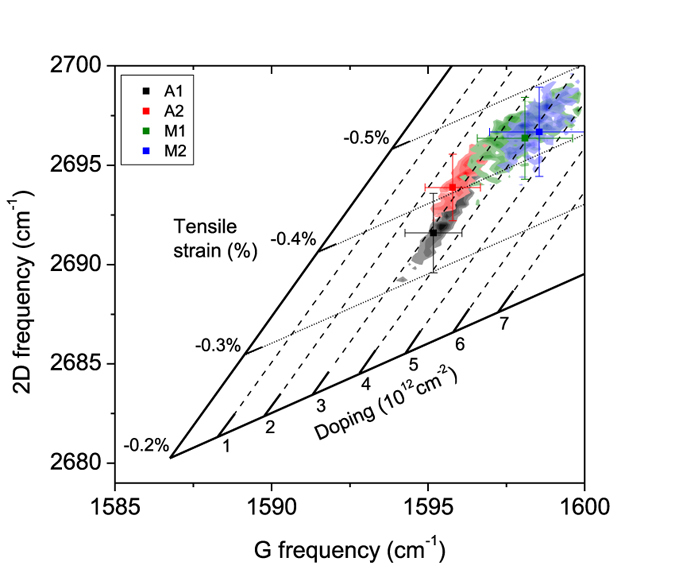
Distribution of the frequency shifts of the Raman G and 2D modes measured in the channel of various GFETs fabricated on automatically (A1 and A2) and manually (M1 and M2) transferred graphene. The center of mass and standard deviations of the distributions are shown by the symbol and the error bars, respectively. The embedded internal axes decouple the doping and tensile strain effects, showing less doping and strain for the devices fabricated on automatically transferred graphene.

Figure 4 shows the correlation between the frequencies 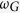 and 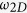 corresponding to the channel area of four GFETs, two devices on automatically transferred graphene and another two on manually transferred graphene. The diagonal evolution of the data distribution is well described when 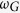 and 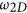 are decomposed in a doping- and stress-related basis, as shown by the internal set of axes (see the Supplementary Information for details on the change of basis). The automatically transferred samples present (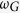, 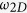) distributions with smaller values than the manually transferred ones, that correspond with a lower amount of both strain and doping. This reduction in strain for the automatically transferred samples is in good agreement with the previously mentioned mechanisms that may enhance strain formation in manually transferred samples. The reduction in doping for the automatically transferred samples is likely related to the more efficient removal of contaminants as discussed previously. The doping ratio between the manually and automatically transferred samples obtained from the optical analysis (Fig. 4) is 1.47, very similar to the value of 1.42 obtained from the electrical analysis (Fig. 3). The small mismatch between the electrically and optically obtained doping levels might be related to the fact that the doping conversion used in the optical decoupling method is based on data from induced charge with liquid gating^36^, whereas doping in the samples of this work is related to adsorbed dopants.

## Conclusion

We have demonstrated a system that automatically transfers graphene to an arbitrary target substrate via the all-fluidic manipulation of the graphene to avoid mechanical damage, strain and contamination and assisted by the combination of capillary and electrostatic repulsion forces between the graphene and its container to center the film respect to the target substrate. In addition to the intrinsic advantages of the automated process (i.e. no handling skills required, good reproducibility, time saving, large throughput, parallel processing, etc.), the automatic system also provides higher quality graphene. GFETs fabricated on the automatically transferred graphene present higher mobility values, lower unintentional doping and strain, and better yields as compared to those fabricated on manually transferred material. The system, initially developed as a lab-scale tool, is fully scalable for industrial applications, allowing the large-scale transfer of graphene films with superior quality to arbitrary substrates.

## Methods

### Self-assembled monolayers surface treatment

The PTFE tubes enclosing the graphene membrane during the automatic transfer process (container tubes) were surface-treated with SAMs^29^, that are able to change the intrinsic hydrophobicity of PTFE and the charge of the surface. First, the container tubes are immersed in Piranha solution (H_2_O_2_:H_2_SO_4_ 1:3 vol.) during 5 minutes and rinsed afterwards thoroughly in DIW. N_2_ drying is performed after each DIW rinsing step. This ensures the cleanliness of the PTFE pieces and reduces their hydrophobicity for the following steps. Next, the container tubes are immersed in an aqueous solution of polystyrene sodium sulfonate (PSS) (2% in mass) for 30 seconds, rinsed in DIW, and dried with N_2_ afterwards. After this, another 30-second immersion in an aqueous solution of polydiallyldimethylammonium chloride (PDDA) (2% in mass), DIW rinse and N_2_ drying are performed. A last PSS treatment as the previous one is performed. Therefore, the final coating is composed of a layered PSS/PDDA/PSS structure.

### GFET processing and electrical characterization

Commercially-available monolayer CVD graphene grown on 25 *μ*m-thick Cu foil has been used as starting material for GFET fabrication. Oxygen plasma has been used for insulating the transistors and Ti/Au (10/200 nm) contacts were deposited by e-beam evaporation. Devices with gate lengths between 10 and 20 *μ*m and widths ranging from 100 to 500 *μ*m have been fabricated. The Si *n*^++^ doped substrate, coated with 300 nm of thermally-grown SiO_2_, has acted as the back-gate. For the characterization, the samples were introduced inside a Janis-CCR10 probe station, which was used for degassing the last residues in graphene at 500 K for 3 hours under vacuum conditions (10^−5^ mbar). After letting the probe station cool down and without breaking vacuum, the current-voltage characteristics for a large number of devices in each sample were measured at room temperature. Back-gate voltage values between −40 and 65 V and a drain-source voltage of 0.2 V were used.

### Raman spectroscopy

A WITec Alpha 300AR Raman confocal microscope has been used for the Raman spectroscopy mapping of the channel area of the GFETs. Raman spectra were obtained in backscattering geometry using a ×50 objective lens (numerical aperture NA = 0.8) in ambient conditions. A 532 nm wavelength laser set to a power of 3 mW was used as excitation source. Typical mapped areas were 110 × 8 *μ*m^2^ in size, scanned with steps of 0.33 *μ*m.

## Additional Information

**How to cite this article**: Boscá, A. *et al.* Automatic graphene transfer system for improved material quality and efficiency. *Sci. Rep.* **6**, 21676; doi: 10.1038/srep21676 (2016).

## Supplementary Material

Supplementary Information

Supplementary video
