# Supplementary material for: Automatic graphene transfer system for improved material quality and efficiency
Source: Sci Rep. 2016 Feb 10;6:21676. doi: 10.1038/srep21676 (PMC4748278; doi:10.1038/srep21676)
Supplement: Supplementary Information [file srep21676-s1.pdf]

Supplementary Information

Automatic graphene transfer system for improved material quality and efficiency

Alberto Boscá, Jorge Pedrós, Javier Martínez, Tomás Palacios and Fernando Calle

System scalability:

To explore the effects and possibilities involved in the system scaling, an adapted prototype has been designed for 4-inch target substrates. Although most of the system parts remain unchanged, the holding structure and the etch beaker have been modified in order to enclose larger samples and target substrates. In Figure S1a the scaled-up system at the begining of the automated process is shown. Figure S1b shows an intermediate state, where the copper has already been etched and the etchant has been partially diluted. In Figure S1c the sample is ready for the final transfer step, where the DIW is depleted and the PMMA/graphene membrane is deposited on top of the 4-inch SiO_2/Si wafer (Figure S1d). The self-centering effect inside the bigger container tube is effective despite the change in dimensions of both sample and target substrate. Therefore the transfer mechanism proves to be easily scalable.

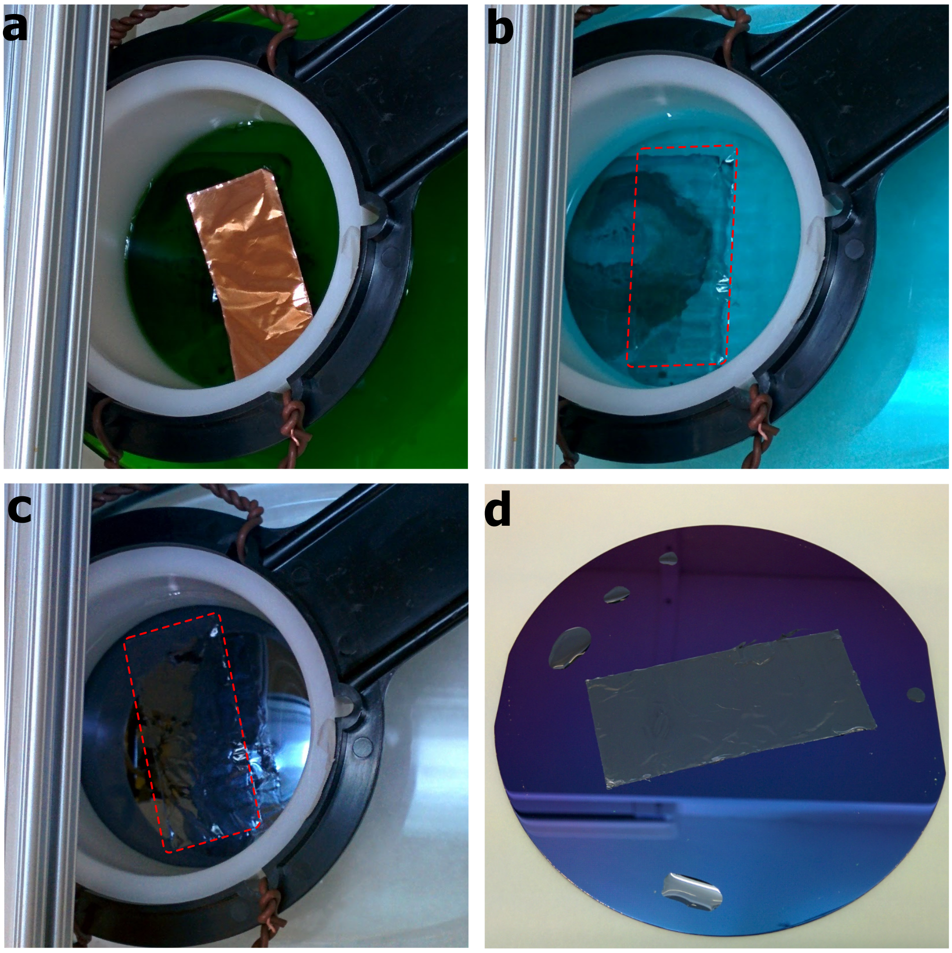

Figure S1: a) Initial, b) intermediate and c) final steps of the automated transfer for 4 inches substrates. d) 4-inch substrate with the 6.5x2.7 cm^2 PMMA/graphene membrane on top.

Ion concentration study:

A potentiostat/galvanostat (Autolab PGSTAT302N) and a 2-electrode Pt cell have been used for measuring the liquid conductivity. The conductivity of ultrapure-water (Millipore Milli-Q type I ultra-pure water) has been measured 100 times over a 2 hour period-time for establishing the deionized water (DIW) conductivity value. The DIW conductivity range centered in $5.50x10^{-2}$ $\mu S \cdot cm^{-1}$ is represented with horizontal blue lines in Figure S2. For measuring the conductivity at every dilution step, an automatic transfer has been performed. The conductivity has been measured 20 times for each dilution step, being the system at the maximum filling level during the measurements. It is extracted from the graph that at least 26 dilution steps are needed to reach the DIW conductivity level.

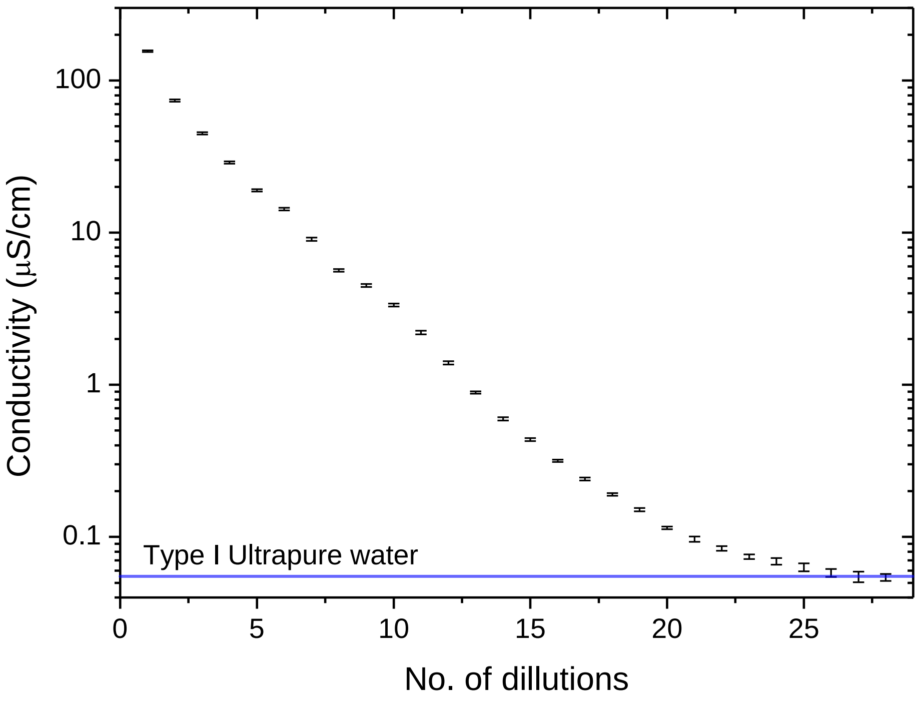

Figure S2: Conductivity evolution for successive dilutions.

Atomic Force Microscopy images:

The AFM images of the surface of the graphene samples show root mean square (RMS) values ranging from 0.89 to 0.95 nm, as shown for example in Figure S3. These values are comparable to those reported in the literature (Table 1).

Table 1: RMS values extracted from the literature using different transfer methods.

Method	Conditions	RMS (nm)	Evaluation Region	Reference
Manual (PMMA)	DIW	3.27	10x10 μm ^{2} area	Reina et al.[4]
Manual (PMMA)	DIW	4.6	5x5 μm ^{2} area	Pirkle et al.[5]
Manual (PMMA)	DIW+ annealing	0.6	5x5 μm ^{2} area	Pirkle et al.[5]
Manual (PMMA)	DIW	4.16	10 μm line scan	Gao et al.[6] (Calculated)
Manual (PMMA)	DIW @ 80 °C	0.75	20 μm line scan	Gao et al.[6] (Calculated)
Manual (PMMA)	IPA	0.34	10 μm line scan	Gao et al.[6] (Calculated)
Manual (PMMA)	DIW	5.06	10 μm line scan	Hallam et al.[7] (Calculated)
Manual (NC[1])	DIW	3.24	8 μm line scan	Hallam et al.[7] (Calculated)
Manual (CAB[2])	DIW	2.44	6 μm line scan	Hallam et al.[7] (Calculated)
Roll to Roll		0.87	30 μm line scan	Bae et al.[8] (Calculated)

[1] Cellulose Nitrate, [2] Cellulose Acetate Butyrate.

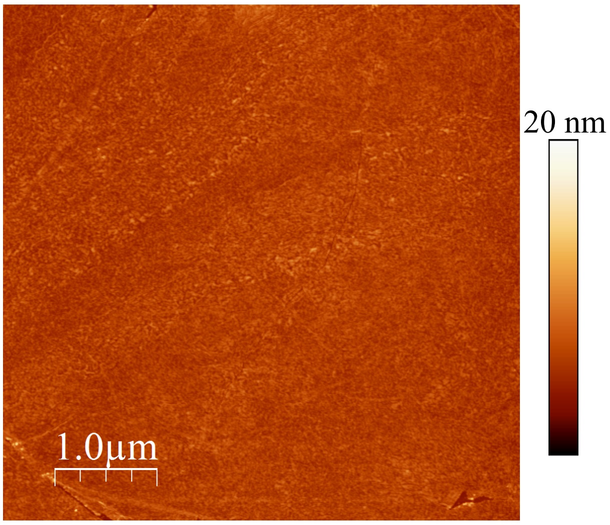

Figure S3: AFM image of a 5x5 μm^2 area of the graphene surface. The RMS is 0.91 nm.

Strain and doping in the manual transfer method:

Figure S4a shows how the abrupt *fishing* step used repeatedly in the manual transfer method to catch the floating PMMA/graphene membrane may raise some extra tensile strain in the samples. Figure S4b shows how during the same *fishing* steps in the manual method an enhanced ion trapping in the graphene surface may occur, as wrinkles are generated around etchant residues that are pressed against the membrane.

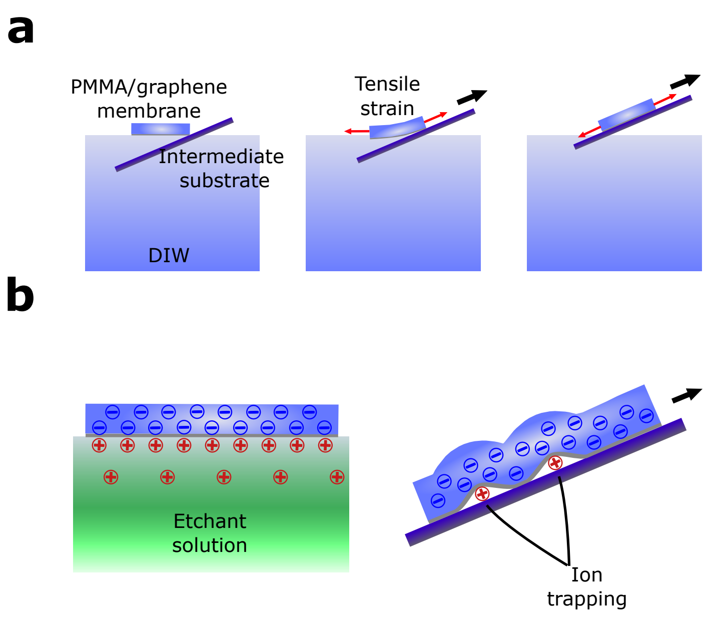

Figure S4: Schematic of the mechanisms producing the extra (a) tensile strain and (b) doping values found using the manual method.

Raman spectroscopy mapping and strain and doping decoupling analysis:

In Figure S5, the Raman spectrum of the commercially-available monolayer ($I_{2D}/I_G > 2$) graphene used is shown.

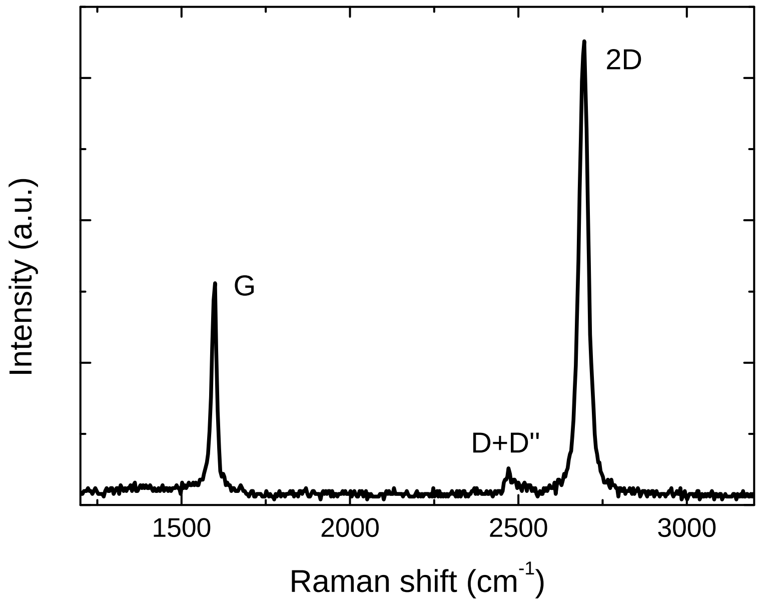

Figure S5: Raman spectrum of the commercially-available graphene used.

Based on the work of Lee *et al.*,[1] the strain and doping-induced shifts on the 2D and G Raman mode frequencies were decomposed. An algebraic change of basis has been performed, where the matrix components have been calculated using literature values. This method implies a linear assumption for the doping, which is found to be valid for the range of values included in this work (as indicated by the consistency with those obtained from the electrical

measurements), but might be inaccurate for doping values of ultra-clean, undoped graphene. As some shift in the 2D mode is related to the laser energy ($\lambda = 532$ nm), the origin reported by Lee *et al.*[1] ($\lambda = 514.5$ nm) has been corrected using a ω_{2D} shift of 88 cm^{-1}/eV.[2]

$$O = (1581.6, 2676.9 + 88 \cdot \Delta E(eV)) = (1581.6, 2669.9) \;\; (cm^{-1}) \tag{1}$$

Two different basis (B_1 and B_2) sharing the same origin point (O from Equation 1) are defined for the frequency space $\{\omega_G, \omega_{2D}\}$ (in cm^{-1}):

$$B_1 = \{\vec{e}_{\Delta\omega_G}, \vec{e}_{\Delta\omega_{2D}}\} = \{(1,0), (0,1)\}, \tag{2}$$

$$B_2 = \{\vec{e}_{\Delta\omega_{Q_f}}, \vec{e}_{\Delta\omega_\varepsilon}\} = \{(0.81, 0.57), (-0.41, -0.91)\}, \tag{3}$$

where B_2 unitary vectors have been calculated using the slope values[1]:

$$\left(\frac{\Delta\omega_{2D}}{\Delta\omega_G}\right)_\varepsilon = 2.2; \quad \left(\frac{\Delta\omega_{2D}}{\Delta\omega_G}\right)_{Q_f} = 0.70. \tag{4}$$

B_1 defines any point $\overrightarrow{OP}$ in terms of the ω_G and ω_{2D} shift from the origin, whereas B_2 defines any point in terms of the 'strain-free' ($\vec{e}_{\Delta\omega_{Q_f}}$) and 'charge-neutral' ($\vec{e}_{\Delta\omega_\varepsilon}$) unit vectors:

$$\overrightarrow{OP} = \Delta\omega_G \cdot \vec{e}_{\Delta\omega_G} + \Delta\omega_{2D} \cdot \vec{e}_{\Delta\omega_{2D}} = \Delta\omega_{Q_f} \cdot \vec{e}_{\Delta\omega_{Q_f}} + \Delta\omega_\varepsilon \cdot \vec{e}_{\Delta\omega_\varepsilon} \tag{5}$$

Therefore, a simple matrix M and its inverse (M^{-1}) can be used for changing from B_1 to B_2 and vice versa:

$$\begin{pmatrix} \Delta\omega_G \\ \Delta\omega_{2D} \end{pmatrix} = M \cdot \begin{pmatrix} \Delta\omega_{Q_f} \\ \Delta\omega_\varepsilon \end{pmatrix} = \begin{pmatrix} 0.81 & -0.41 \\ 0.57 & -0.91 \end{pmatrix} \cdot \begin{pmatrix} \Delta\omega_{Q_f} \\ \Delta\omega_\varepsilon \end{pmatrix} \tag{6}$$

$$\begin{pmatrix} \Delta\omega_{Q_f} \\ \Delta\omega_\varepsilon \end{pmatrix} = M^{-1} \cdot \begin{pmatrix} \Delta\omega_G \\ \Delta\omega_{2D} \end{pmatrix} \tag{7}$$

Then, for any point expressed in the B_1 basis ($\Delta\omega_G, \Delta\omega_{2D}$), the frequency shift can be expressed in terms of the B_2 basis ($\Delta\omega_{Q_f}, \Delta\omega_\varepsilon$). Thus, the frequency displacement separated for doping ($\Delta\omega_G(Q_f), \Delta\omega_{2D}(Q_f)$) and strain ($\Delta\omega_G(\varepsilon), \Delta\omega_{2D}(\varepsilon)$) can be obtained by changing back to the B_1 basis each vector component expressed in the B_2 basis alone:

$$\begin{pmatrix} \Delta\omega_G(Q_f) \\ \Delta\omega_{2D}(Q_f) \end{pmatrix} = M \cdot \begin{pmatrix} \Delta\omega_{Q_f} \\ 0 \end{pmatrix} \tag{8}$$

$$\begin{pmatrix} \Delta\omega_G(\varepsilon) \\ \Delta\omega_{2D}(\varepsilon) \end{pmatrix} = M \cdot \begin{pmatrix} 0 \\ \Delta\omega_\varepsilon \end{pmatrix} \tag{9}$$

With these frequency shift values and the sensistivity values $d\omega_{2D}(Q_f)/dQ_f = -1.04$ ($\text{cm}^{-1}/10^{12}$ cm^{-2}), $d\omega_G(\varepsilon)/d\varepsilon = -23.5$ ($\text{cm}^{-1}/\%$) extracted from the literature[1,3], the doping and strain distributions can be calculated for all the Raman spectra:

$$|Q_f|/q = \frac{\Delta\omega_{2D}(Q_f)}{d\omega_{2D}(Q_f)/dQ_f} \quad (10^{12}\,\text{cm}^{-2}) \tag{10}$$

$$\varepsilon = \frac{\Delta\omega_G(\varepsilon)}{d\omega_G(\varepsilon)/d\varepsilon} \qquad (\%) \tag{11}$$

This method has been applied to the Raman data obtained from the mapping of several GFET channel areas. Figure S6 presents the histograms for two of them.

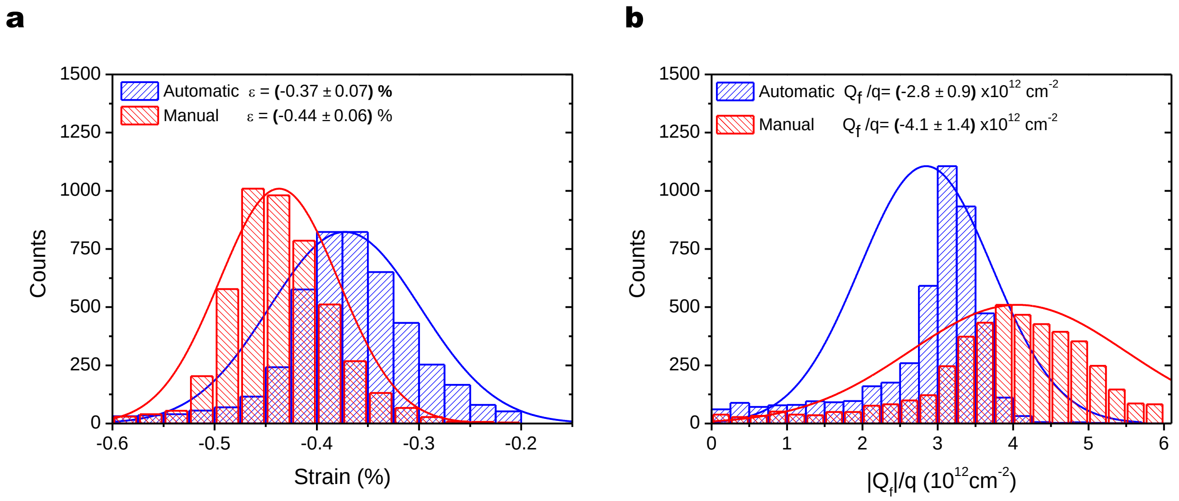

Figure S6: Histograms of automatically and manually transferred device-channel data for (a) strain and (b) doping extracted from Raman spectroscopy measurements.

[1] Lee, J. E., Ahn, G., Shim, J., Lee, Y. S., and Ryu, S. *Nature Communications* **3**(May), 1024 (2012).

[2] Mafra, D. L., Samsonidze, G., Malard, L. M., Elias, D. C., Brant, J. C., Plentz, F., Alves, E. S., and Pimenta, M. A. *Physical Review B* **76**(23), 233407 (2007).

[3] Das, A., Pisana, S., Chakraborty, B., Piscanec, S., Saha, S. K., Waghmare, U. V., Novoselov, K. S., Krishnamurthy, H. R., Geim, A. K., Ferrari, A. C., and Sood, A. K. *Nature Nanotechnology* **3**(4), 210 (2008).

[4] Reina, A., Jia, X., Ho, J., Nezich, D., Son, H., Bulovic, V., Dresselhaus, M. S., and Kong, J. *Nano Letters* **9**(1), 30 (2009).

[5] Pirkle, A., Chan, J., Venugopal, A., Hinojos, D., Magnuson, C. W., McDonnell, S., Colombo, L., Vogel, E. M., Ruoff, R. S., and Wallace, R. M. *Applied Physics Letters* **99**(12), 122108 (2011).

[6] Gao, L., Ni, G.-X., Liu, Y., Liu, B., Castro Neto, A. H., and Loh, K. P. *Nature* **505**(7482), 190 (2013).

[7] Hallam, T., Berner, N. C., Yim, C., and Duesberg, G. S. *Advanced Materials Interfaces* **1**(6), 1400115 (2014).

[8] Bae, S., Kim, H., Lee, Y., Xu, X., Park, J.-S., Zheng, Y., Balakrishnan, J., Lei, T., Ri Kim, H., Song, Y. I., Kim, Y.-J., Kim, K. S., Özyilmaz, B., Ahn, J.-H., Hong, B. H., and Iijima, S. *Nature Nanotechnology* **5**(8), 574 (2010).
